# Epigenetic mechanisms of alveolar macrophage activation in chemical-induced acute lung injury

**DOI:** 10.3389/fimmu.2024.1488913

**Published:** 2024-11-08

**Authors:** Shama Ahmad, Wesam Nasser, Aftab Ahmad

**Affiliations:** Anesthesiology and Perioperative Medicine, University of Alabama at Birmingham, Birmingham, AL, United States

**Keywords:** macrophage, lung, inhaled, chemical, halogen, epigenetics

## Abstract

Airways, alveoli and the pulmonary tissues are the most vulnerable to the external environment including occasional deliberate or accidental exposure to highly toxic chemical gases. However, there are many effective protective mechanisms that maintain the integrity of the pulmonary tissues and preserve lung function. Alveolar macrophages form the first line of defense against any pathogen or chemical/reactant that crosses the airway mucociliary barrier and reaches the alveolar region. Resident alveolar macrophages are activated or circulating monocytes infiltrate the airspace to contribute towards inflammatory or reparative responses. Studies on response of alveolar macrophages to noxious stimuli are rapidly emerging and alveolar macrophage are also being sought as therapeutic target. Here such studies have been reviewed and put together for a better understanding of the role pulmonary macrophages in general and alveolar macrophage in particular play in the pathogenesis of disease caused by chemical induced acute lung injury.

## 1 Introduction

**Figure 1:**
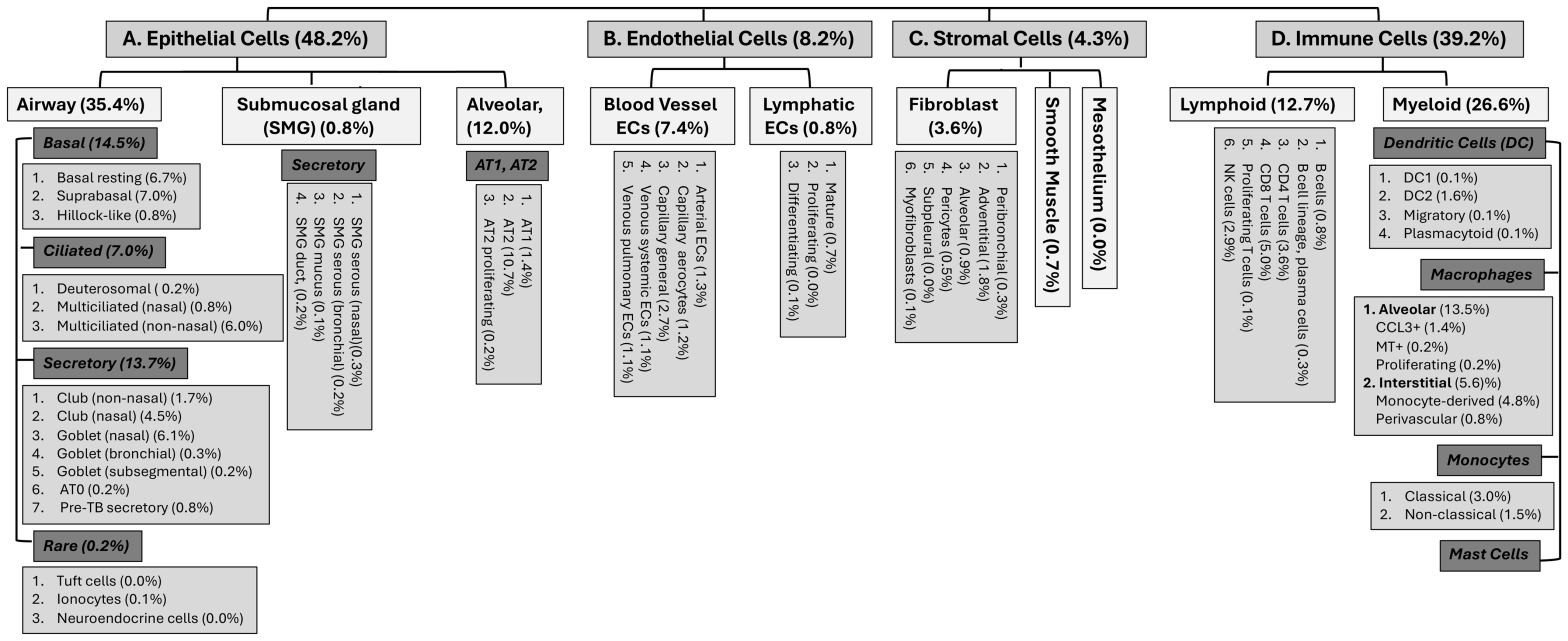
Different cell types and sub-types and their potential distributions in the human pulmonary tissues (2, 3).

The respiratory system is at the frontline of constant encounter by environmental components such as non-toxic or toxic environmental gases and particulates. These can include occasional massive exposures to noxious chemical vapors or gases during accidental/occupational spills or deliberately released poisonous chemicals during terroristic attacks or wars. The type of chemical, its concentration and duration of exposure determine the extent of airway damage, edema, activation of immune cells and inflammation that may often lead to respiratory dysfunction and death. Various pulmonary cells including those of airway and alveolar epithelium, interact with and respond to such toxicants via a cascade of events that include sloughing of epithelial cells, loss of alveolar capillary membrane integrity, and subsequent activation and infiltration of immune cells. Human lungs were originally thought to be composed of about 40 different types of cells (1–4). Emerging technologies like those of single cell sequencing have transformed this information and according to currently published findings, pulmonary tissues were noted to have about 61-62 cell types which may change their identity with altered respiratory health conditions (**Figure 1**) (2). Pulmonary macrophages are the most abundant myeloid cells of the immune system which are the primary controllers of both innate and adaptive immunity and attain several phenotypes to respond to a wide variety of stimuli/insults including environmental pollutants, pathogens (microbes such as fungi, bacteria, virus, their products etc.) or other inhaled toxicants/threat agents (5–13).

Inhaled stimuli such as the toxic vapors or gases induce an immune response which is initiated by both macrophages and the airway epithelium, however the macrophages produce the most potent local proinflammatory response in the lungs which subsequently results in the systemic response (11, 14, 15). Previous studies have shown that the lung microenvironment is important in shaping the distinct transcriptional and epigenetic landscape of the cellular identity and function of resident macrophages (16, 17). Macrophages are the first kind of immune cells that appear during embryogenesis and are essential for early stages of organism development (18). Two types of macrophages populate the lung the a) alveolar macrophage and b) the interstitial macrophage. Alveolar macrophages (AMs) and interstitial macrophages (IMs) reside in different anatomical compartments in the lungs. IMs are monocyte derived uniform cell population that reside in the interstitium (the space between alveolar and vascular endothelium) and are often found to be associated with the airways, nerves and vessels (**Figure 1**). They are less well studied and have been implicated in maintenance of lung homeostasis and in prevention of immune-mediated allergic airway inflammation (19). Macrophages can sense the danger signals from their microenvironment via specific receptors such as the pattern recognition receptors. Both AMs and IMs are activated by various stimuli to polarize and form activated (M1 or M2) macrophages that determine the progression of acute lung injury (20). The nomenclature of such activated macrophage subsets is dynamic and has seen many revisions and now considered too complex with overlapping characteristics, to be defined by these two terms (21). In this review we will cover the mechanisms by which toxic inhaled chemicals and gases affect the alveolar macrophages.

### 1.1 Alveolar macrophages

Alveolar macrophages (AMs) have been shown via lineage tracing studies to originate from the embryonic precursors that populate the alveolar space soon after birth (22). They are the major macrophage population found attached on the epithelial surface, where they not only replicate and self-maintain but also protect the gas exchange function and barrier immunity of the lung. The high self-replicating ability, development, maintenance and function of AMs was shown to be dependent on the neutrophil derived 12-HETE and type II alveolar epithelial cell derived GM-CSF (22, 23). Once damaged or depleted, AMs can be restored by circulating monocytes that are often referred to as monocyte derived alveolar macrophages to distinguish them from the regular tissue resident AMs (9).

#### 1.1.1 Alveolar macrophage identifying markers

Macrophage phenotypes are identified by a combination of multiple markers that enable understanding of their heterogeneity and plasticity in reference to their microenvironment (21, 24, 25). The high degree of diversity observed in the pulmonary resident macrophages (AMs and IMs) is due to their different activation states and contribution of infiltrating monocytes to their populations (**Table 1**) (25, 62). At steady state alveolar macrophages are maintained by epithelial GM-CSF and TGF β (63). When injury occurs, monocytes are recruited to the alveolar lumen and interstitium and develop into the activated monocyte derived macrophages that may further contribute to damage by secreting cytokines or perform reparative function. Alveolar macrophages have been shown to interact with the monocyte derived macrophages and other lymphoid cells to modulate their function (64).

**Table 1:** Common pulmonary (alveolar) macrophage markers utilized for their identification.

Name/Expression	Mouse/Human	Macrophage type	Function	Reference
F4/80 (pan marker)	Mouse	Macrophages	Cell surface antigen of mononuclear phagocytic cells	(26)
CD68 (pan marker)	Human/Mouse	Macrophages	Cell surface antigen and promotes phagocytosis	(27, 28)
Mer/TK	Mouse	Alveolar macrophage	Clearance of apoptotic cells	(29–31)
CD169	Human/Mouse	Alveolar and Interstitial macrophage	Cell surface lectin that promotes immune response	(21, 32, 33)
Siglec F/CD170 (high)	Mouse	Alveolar macrophages	Cell surface lectin that promotes adhesion and immune response	(21)
CD10 (MME)	Human	Alveolar macrophages, monocyte derived macrophages	Membrane metal loendopeptidase, negatively regulates peptide hormones	(34–36)
CD206 (high)	Human	Alveolar macrophages (activated AMs)	A pattern recognition receptor and plays a role in immune response	(34, 37)
CD11c (high)	Human/mouse	Alveolar macrophages	Integrin molecule that enables adhesion to other cells	(34, 38)
CD86 (high)	Human/Mouse	Alveolar macrophages	Stimulatory molecules involved in adaptive immunity	(34, 39)
CD88	Human/Mouse	Alveolar macrophages (activated)	A complement receptor (C5a) that modulates inflammatory response	(40, 41)
CD80 (high)	Human	Alveolar macrophages (activated)	An inducible costimulatory molecule involved in immune activation	(39)
CD163 (high)	Human	Alveolar macrophages (activated)	An anti-inflammatory molecule involved in integrin and hemoxygenase production	(34, 42)
CD141	Human	Alveolar macrophages	Also called thrombomodulin and plays a role in anticoagulation system	(43)
CD64 (high)	Human/Mouse	Alveolar macrophages	An FC Gamma receptor and triggers immune responses upon binding to IgG	(8, 44)
HLADR	Human	Alveolar macrophages	MHCII cell surface receptor, initiates immune response	(45, 46)
MHCII	Mouse	Alveolar macrophages	Antigen processing and presentation, initiates immune response	(47, 48)
CD40	Human/Mouse	Alveolar macrophages (activated)	Mediates proinflammatory response	(49, 50)
MARCO	Human/Mouse	Alveolar macrophages	Host defense, ingestion and opsonization of particulates	(21, 51)
CD43	Human/Mouse	Alveolar macrophages	Cell adhesion, migration and signaling	(52–54)
CD11b (high)	Human	Alveolar macrophages and interstitial macrophages (activated)	Mediation of inflammation	(21, 38)
CD36	Human/Mouse	Alveolar macrophages (activated)	Promotes inflammatory response and phagocytosis, lipid uptake	(55, 56)
SPP1/Osteopontin (high)	Human/Mouse	Alveolar and Interstitial macrophages (activated)	Promotes fibroblast cell attachment, migration and proliferation	(21, 57, 58)
CX3CR1	Human/Mouse	Monocytes, macrophages dendritic cells, T cells, NK cells etc	Mediate chemotaxis of immune cells	(21, 59)
FOLR2	Human/Mouse	Interstitial macrophages	Internalize folate and increase cell delivery to promote proliferation.	(21)
C1Q	Human/Mouse	Alveolar and Interstitial macrophages	Binding and ingestion of opsonized targets, resolution of inflammation	(21) (60) (61)

Many cluster of differentiation (CD) markers or cell surface molecules on the macrophages enable their identification within the pulmonary tissues of humans and mice (**Table 1**). Alveolar macrophages under normal conditions can be identified by the presence of CD11c and interstitial macrophages by CD11b on their surface (21). CD11c, CD169 and MARCO are expressed by the alveolar macrophages of both mice and humans (24, 65). Additional species-specific (human and mice) markers are utilized to identify the alveolar macrophages (**Table 1**). Although, many different (common or unique) macrophage markers have been identified in rodent and human tissues very little information is found for other mammals (66). In alveolar macrophage samples from healthy humans of different geographical areas, high expression of CD64, CD80, CD86, CD163 and CD206 was observed although normally these markers are associated with polarized M1/M2 macrophages (67). Some of these markers (CD206) are altered by the lung microenvironment such as surfactant protein content (68). On the other hand, some markers (e.g. MARCO) may act as target of therapy for alleviating pulmonary fibrosis (69). In general, the resident alveolar macrophages are characterized by F4/80^+^, CD64^+^, MerTK^+^, SiglecF^hi^, CD11c^hi^, CD11b^lo^ and CD206^hi^ markers. The monocyte derived alveolar macrophages demonstrate F4/80^+^, CD64^+^, MerTK^+^, SiglecF^-^, CD11c^-^, CD11b^hi^ and CD206^lo^ markers and is generally proinflammatory and involved with pathogen phagocytosis and cytokine production. There are two kinds of interstitial macrophage populations a) MHCII^hi^ Lyve^lo^ with F4/80^+^, CD64^+^, MerTK^+^, SiglecF^-^, CD11c^-^, CD11b^hi^, CD206^lo^ and CX3CR1^hi^ markers that represents the antigen presenting and proinflammatory macrophage population and b) MHCII^lo^ Lyve^hi^ with F4/80^+^, CD64^+^, MerTK^+^, SiglecF^-^, CD11c^-^, CD11b^hi^, CD206^hi^ and CX3CR1^lo^ markers, which represents the wound healing and tissue repair population (62). However, antibody specificity, origin and detection method can greatly affect the identity of the cells (70, 71).

#### 1.1.2 Alveolar macrophages and inhaled chemical-induced acute lung injury

Alveolar macrophages are one of the first cells to encounter the inhaled chemicals. As mentioned earlier toxic vapors or gas initiate responses by both macrophages and the airway epithelial cells, however the macrophages produce the most potent local proinflammatory response in the lungs which subsequently results in the systemic response (11, 14, 15). Toxic chemicals and environmental agents may not only be affecting the macrophage function but may often lead to their decreased clearance and subsequent accumulation to cause long term consequences (72). Adverse effects of inhaled pharmaceutical agents delivered for therapy are not covered here but can be read elsewhere (73, 74). Inhaled anesthetics also modulate the macrophage function and may alter pulmonary disease outcomes (75–78). Similarly, AMs contribute to the inflammatory response induced by cigarette smoke and environmental pollutants (14, 79–81). Common toxic environmental pollutants and gases like sulfur dioxide damage and reduce AM function (82–88). Immune cell development and their responses are highly susceptible to environmental factors.

Many highly toxic chemicals such as sulfur mustard, chlorine gas, ammonia, and phosgene exert their harmful effects through direct toxicity to the pulmonary epithelium and endothelium leading to cell death, loss of barrier function, and increased permeability (89, 90). Reactive oxygen species (ROS) generated either directly by the toxic agent or because of cellular injury also play a significant role in the pathophysiology of chemical-induced lung damage (91). The imbalance between ROS and the antioxidant defenses leads to oxidative stress damaging cellular proteins, lipids, and DNA (92). Chemical exposure also triggers a robust inflammatory response, characterized by the recruitment of immune cells, including macrophages and neutrophils, to the lung (93). While initially aimed at clearing the injury, persistent inflammation can lead to further tissue damage and fibrosis (94). Macrophages play an essential role as critical regulators in the early response to chemical injury, repair, or progression towards fibrosis. When exposed to noxious chemicals, macrophages are among the first responders, releasing pro-inflammatory cytokines and chemokines that mediate the acute inflammatory phase (95). However, dysregulated activation of macrophages can further exacerbate tissue damage and lead to fibrosis through the release of various profibrotic factors (96).

Alveolar macrophages are strategically situated at the tissue-air interface where they play important role in regulating the pulmonary immune response (97). This has been demonstrated by several studies where depletion of alveolar macrophages caused increased immune response to particulate antigens (98), reduced neutrophil influx (99), decreased clearance of antigens (100) and increased lung injury (101). Depletion of macrophages on the other hand facilitated efficient absorption of therapeutic macromolecules (102) and reduced inflammatory response after acid and radiation-induced lung injury in experimental models (103, 104). Currently, studies to clarify the role of monocyte derived circulating macrophages and the resident alveolar macrophages, where monocytes/macrophages were depleted from circulation demonstrated protection from adverse lung conditions resulting from inhaled endotoxic exposures (105). Thus, AMs are the primary immune cells of the lung at steady state where their function is to dampen inflammatory response, but this immune balance can be easily disturbed by perturbations in surrounding microenvironment. In the lungs, the AMs are constantly bathed by the surfactant containing airway lining fluid layer. Inhaled noxious substances can also damage the airway lining fluid and destroy or inhibit the factors that promote macrophage function (106). The outcome of tissue responses to such toxic agents depends on the balance of the mediators produced by the normal quiescent alveolar macrophages and those from the activated alveolar macrophages exposed to the toxic compounds (107).

##### 1.1.2.1 Inhaled halogens and phosgene

Elemental halogens such as chlorine (Cl_2_) and bromine (Br_2_) and phosgene (COCl_2_) are common toxic industrial chemicals and have been used historically in warfare and currently in various armed conflicts (108–111). They are highly reactive oxidizing and corrosive agents and cause acute lung injury (ALI), acute respiratory distress syndrome (ARDS) and even cardiac damage to the exposed individuals when present in high dose and prolonged duration (109, 112–122). One of the earliest case report of bronchial brushings from patients exposed accidently to chlorine demonstrated increased presence of non-pigmented alveolar macrophages 5 days after exposure (123). A dose dependent increase in bronchoalveolar lavage fluid alveolar (BALF) macrophage content was observed at 24h post chlorine exposure in mice (124). Increased inducible nitric oxide and 3-nitrotyrosine content was detected in the macrophages by these authors. Chlorine exposure causes TRPV activation which can result in formation of ROS and NO leading to peroxynitrite formation and tyrosine nitrosylation of proteins (125–127). In another study using relatively lower concentrations of chlorine the macrophage content was unaltered in the BALF up to 48h post exposure (128). These investigators did observe increase in the genes (e.g. Arg1) related to alternative macrophage activation. Damage to the airway lining fluid as demonstrated by loss of surface-active function by chlorine exposure was also demonstrated in this study (128). Chlorine exposure did not alter the number of resident macrophages and anti-inflammatory macrophages in the BALF collected 24h after exposure (129). However, the number of COX-2 or iNOS expressing proinflammatory macrophages were increased in this study. With repeated chlorine exposure a pulmonary adaptation to oxidative stress was observed which could be related to a specific alveolar macrophage population which was dependent on TGF-β and prostaglandin E2 (130). Hemeoxygenase dependent increase in pulmonary macrophages 24h after bromine exposure were recently reported (131–133). Phosgene exposure in mice reduced the BALF macrophage content when evaluated 24h after exposure (134). This could be related to the interaction of phosgene with surfactant and the role AMs play in endocytosis of dysfunctional surfactant proteins and subsequent efferocytosis of these overloaded AMs (135). Not surprisingly, inhibition of phosgene-induced AM galectin-3 production reduced alveolar epithelial cell death and lung damage (136).

##### 1.1.2.2 Inhaled mustard vesicants

Exposure to vesicants like sulfur mustard (SM) and nitrogen mustard (NM) activate the pulmonary macrophages by ensuing inflammation which furthers the tissue damage by production of ROS and proinflammatory mediators (137–140). Dermally applied vesicants also cause increased macrophage content in the lungs of mice after exposure (141, 142). A nonsignificant decrease in BALF macrophage content after exposure to half mustard (2-chloroethyl ethyl sulfide, CEES) was reported in rats 18h after exposure (143). Inhaled nitrogen mustard caused increased inflammatory cytokine (TNFα) production and infiltration of CD11b+ macrophages in the lungs of exposed mice 3 days after exposure (144). The resident alveolar macrophages decreased upon NM treatment and were replaced by the infiltrating proinflammatory CD11b^+^ macrophages of M1 phenotype that matured later into profibrotic M2 macrophages suggesting a role of alveolar macrophages in the pathogenesis of NM injury (145). Treatment with anti-TNFα antibody not only reduced the macrophage infiltration in the lungs but also reduced injury, inflammation and subsequent fibrosis in this model and in similar SM model (144, 146, 147). Macrophage derived TNFα has been described as a major pathway in vesicant induced lung injury (148, 149). NM induced macrophage activation and lung injury could also be mitigated by antioxidants like N-acetyl cysteine (NAC) and surfactant protein D administration (137, 150). NAC was also protective against CEES induced lung injury in a guinea pig model (151). A role of histones, miRNA and histone acetylase and deacetylase in the phenotype switching of the alveolar macrophages was demonstrated in this model (152, 153). Transcriptional profiling of the early inflammatory phase and later profibrotic/resolution phase of alveolar macrophages from NM treated animals identified cytokine genes involved in cell migration and significant enrichment of canonical pathways related to STAT3 and NFκB signaling (154). Farnesoid X receptor (FXR) that regulates lipid homeostasis and inflammation was shown to limit alveolar macrophage inflammatory response in a mouse model of IT administered NM (155). Many studies with cutaneous or inhaled vesicant demonstrate increased HMGB1 in the BALF (121, 141, 156–159). HMGB1 causes increase in IL6 and TNF-α that switch the macrophage phenotype (160). Macrophage polarization, polarization phenotypes and their intermediates can be potential new targets to reduce inflammatory responses and tissue injury caused by inhaled vesicants and other toxic stimuli (161, 162).

##### 1.1.2.3 Inhaled ammonia and acids

Ammonia is a highly reactive irritant gas and a toxic industrial chemical, which quickly forms caustic ammonium hydroxide on moist surfaces. Exposure to ammonia causes burns on skin and acute respiratory tract injury, pulmonary edema and respiratory failure. The survivors have long term pulmonary complications and develop bronchiectasis, AHR, BO, COPD (163–166). Long term ammonia exposure in occupational settings causes lower airway diseases and ILD which sometimes even need lung transplantation (167). High dose ammonia inhalation can be lethal and the extent of lung injury and damage is a predictor of fatal outcome (168, 169). Intratracheal ammonia administration in animal models causes severe lung injury, respiratory acidosis and alveolar and interstitial damage (170–172). Increased infiltration of pulmonary macrophages was observed on day 7 after exposure, although neutrophils and inflammatory mediators were significantly increased at day 1 postexposure (171). Others also reported increased inflammatory cells in animals exposed to various concentration of inhaled ammonia (172, 173). Sensitivity of various mice strains to pulmonary toxicity by ammonia has also been demonstrated and many candidate genes were identified that determined the susceptibility to ammonia (174). Exposure to ammonia causes oxidative stress and increased mRNA levels of glutathione peroxidase, COX-2, iNOS, TNF-α and TGF-β (175–177). Whether the TNF-α is macrophage derived in this model is unknown but genes related to macrophage infiltration were increased upon ammonia exposure in a swine model (176).

Exposure to acids such as sulfuric acid may alter the clearance from the alveolar region by affecting the alveolar macrophage function (106, 178). HCl administration in mice causes both acute and chronic lung injury and HSP70 and HSP90 were shown to play regulatory role in causing endothelial barrier disruption and dysfunction (179–186). HCl administration increased BAL macrophages and proinflammatory cytokines like TNF-α, MCP-1 and IL-1β (187, 188). HCl administration into rabbit trachea caused increased neutrophil influx in the lungs and reduced alveolar macrophage adherence function (189). Acid induced lung injury and fibrosis was alleviated by a TLPQ-21 derivative which is an activator of alveolar macrophage function (190). Thus, alveolar macrophages are activated upon acute exposure to toxic chemicals and acids causing tissue damage and inflammation which may eventually lead to chronic effects including fibrosis.

#### 1.1.3 Epigenetic mechanisms driving macrophage functions

Macrophages are pivotal to the innate immune system, especially within the lung environment, where they are primarily responsible for detecting, engulfing, and destroying pathogens through phagocytosis (191). They also secrete various cytokines and chemokines that mediate inflammation and recruit other immune cells to infection sites, crucial for controlling infection spread and initiating tissue repair processes (192). Macrophages display remarkable versatility in their activation; they respond to a variety of cytokines and pathogen-associated signals, which can drastically alter their behavior and function, adapting to the needs of the host defense and repair mechanisms (193). Macrophages are important in initiating immune responses through their role as antigen-presenting cells, which is critical for linking the innate and adaptive sides of the immune system (194).

Phenotypic plasticity results in macrophages that can convert from one functional phenotype to another in response to local microenvironment signals (195). This plasticity allows them to adopt various roles, from pro-inflammatory (activated, M1) phenotypes, which are essential during the initial phases of infection and inflammation, to anti-inflammatory and tissue repairing resident (M2) phenotype, crucial for resolution of inflammation and tissue healing (196). Macrophages switch between these phenotypes under the influence of environmental cues and cytokines, a process that is essential for the balanced immune response required to resolve infections while minimizing tissue damage efficiently (197). The plasticity of macrophages in function is such a key point that significantly assists in understanding their complete involvement in either disease progression or resolution within the lung. The ability of macrophages to switch phenotypes is not only essential in the resolution of different phases of diseases but also provides potential therapeutic targets through manipulating these transitions in diseases such as asthma and COPD (65, 198).

Epigenetic regulations are essential in controlling the functions of cells of innate immunity, including macrophages. Epigenetic changes to genes have the ability to change their expression without altering the sequence, significantly impacting the actions of macrophages and, thus, affecting the overall immune response (199). Genes are generally repressed by DNA methylation, which is crucial for the differentiation and functioning of cells. DNA methylation has been shown to impact the expression of specific cytokines and other proteins in macrophages. Abnormal methylation patterns are related to changes in macrophage activities, which influence the immune response to microbes and injury (200). Histone modifications, such as acetylation and methylation, can act to either promote or inhibit gene expression. Histone acetylation in macrophages is necessary for the transcriptional activation of inflammatory genes after infection (201). At the same time, the expression of these genes can be inhibited by histone deacetylases (HDCAs), enabling dynamic suppression of inflammatory responses. Histone lactylation is another fairly recent concept linking metabolic changes to epigenetic modifications in cells including macrophages (6). Histone lactylation was shown to affect the polarization of macrophages and release of lactylated DAMPS like HMGB1 from such macrophages promoted endothelial permeability and pathogenesis of sepsis (202, 203).

Non-coding RNAs, including microRNAs (miRNAs) and long non-coding RNAs (lncRNAs), also play a significant role in regulating gene expression in innate immunity. miRNAs can fine-tune the immune response by targeting mRNA transcripts for degradation or inhibiting their translation. miR-155 enhances the inflammatory response by modulating pathways that affect cytokine production in macrophages (204). lncRNAs contribute to the regulation of immune gene expression by interacting with chromatin-modifying complexes, thereby influencing the epigenetic landscape of immune cells (65, 205). These miRNAs and lncRNAs are often packaged in extracellular vesicles (EVs) released from macrophages and can influence the course of injury or disease process.

#### 1.1.4 Alveolar macrophages and epigenetic mechanisms in inhaled chemical injury

Extracellular vesicles (EVs) are cell derived membranous structures that are shed in the extracellular microenvironment that is a critical component of the epigenetic landscape where inflammatory signaling including those of the inflammatory cells/activated macrophages establish crosstalk with chromatin leading to transcription of inflammatory genes (65, 206). EVs and their cargoes are generated by multiple cell types including the alveolar macrophages after exposure to toxicants (207). The cargoes of EVs and their cargoes generated from exposure to toxic gases can potentially be transferred to other cells to promote their effects. EVs especially those from the macrophages mediate epigenetic pathways that regulate injurious and inflammatory responses of inhaled toxicants (208–211). It was demonstrated that the imbalance of histone acetylase and deacetylase contributes to lung macrophage activation following inhaled nitrogen mustard exposure (152). Additional alterations such as DNA methylation could be contributed by metabolic changes during macrophage activation by inhaled toxicants (212). Additional epigenetic mechanisms may be involved in general lung injury caused by inhaled poisonous gases like sulfur mustard (213).

#### 1.1.5 Alveolar macrophages activating pathways and therapeutic targets for chemical induced ALI

Resident alveolar macrophages are highly influenced by their local pulmonary microenvironment which includes the airspace and the vasculature. Despite their protective roles in normal conditions, once activated during injury they play diverse roles in both initiating and driving inflammatory pathways post chemical exposure in the lung making them the ideal therapeutic target (214). Alveolar macrophages have several attributes that make them attractive and effective therapeutic targets viz; their position at the airway-tissue interface, they mediate early innate immune response, availability of inhalable products designed to target them and their long life and immune training (215, 216). Chlorine exposure increased activated proinflammatory alveolar macrophages that expressed COX-2 and iNOS (129, 217). Treatment with corticosteroids like dexamethasone or budesonide reduced inflammation and fibrosis in the lungs of chlorine exposed mice (217, 218). Chlorine-induced airway hyperreactivity was reversed by inhibition of inducible nitric oxide synthase (iNOS) which was potentially contributed by resident alveolar macrophages (124). Phosgene exposure increased inflammatory cytokines like IL-6 and impaired macrophage function and reduced viral clearance in influenza-infected rats (219, 220). Single cell RNA sequencing studies revealed that macrophages and macrophage proliferating cells were prominent clusters of cells in the BALF of chemical (phosgene) induced acute lung injury in rats (221, 222). Phosgene exposure enhanced galectin 3 expression on alveolar macrophages causing enhanced interaction with alveolar epithelial cells leading to membrane damage and death. Galectin 3 inhibition or elimination of alveolar macrophages protected the alveolar epithelial cells and reduced alveolar damage after phosgene exposure (136). Another important aspect of alveolar macrophage activation is formation of foamy or lipid laden macrophages (LLMs) (223, 224). The formation of LLMs is enhanced in inflammation where lipid accumulation compromises the macrophage function. Dysregulated lipid metabolism most commonly due to oxidative stress during lung injury contributes to LLM formation and can be an important therapeutic target. Accordingly, it was shown that antioxidants like NAC reduced LLM formation (225). Therefore, it is critical to explore the interactions of macrophages and other cells and to understand the mechanisms underlying macrophage phenotype development in order to evaluate therapies for diseases associated with acute lung injury caused by chemical exposures.

## 2 Discussion

Alveolar macrophages have important roles in host defense against environmental pathogens, particulates and toxic chemicals that enter through the airways into the alveolar space. Macrophages play an essential role as key regulators in the early response to chemical injury, influencing repair, or progression towards fibrosis. When exposed to noxious chemicals, macrophages are among the first responders, releasing pro-inflammatory cytokines and chemokines that mediate the acute inflammatory phase (39). However, dysregulated activation of macrophages can further exacerbate tissue damage and lead to fibrosis through the release of various profibrotic factors (96). DNA methylation, histone modification, and noncoding RNAs are some of the key epigenetic mechanisms that have been demonstrated to have a considerable impact on the macrophage’s response to chemicals. These mechansims along with others will help bridge the gap in understanding that exists in the development of chronic lung diseases resulting from chemical exposure-induced acute lung injury.
